# Comparison of Quality of Life between Patients Undergoing Submental Endoscopic Thyroidectomy and Conventional Thyroidectomy: A Prospective Controlled Clinical Trial

**DOI:** 10.3390/jcm11164802

**Published:** 2022-08-17

**Authors:** Patorn Piromchai

**Affiliations:** Department of Otorhinolaryngology, Faculty of Medicine, Khon Kaen University, Khon Kaen 40002, Thailand; patorn@kku.ac.th

**Keywords:** endoscopic thyroidectomy, submental, approach, quality of life

## Abstract

The objectives of this study were to compare the surgical outcomes and quality of life between patients undergoing submental endoscopic thyroidectomy and those undergoing conventional thyroidectomy. The surgical outcomes and quality of life were recorded. Forty-eight patients were included in the study. Their ages ranged from 20 to 60 years. All patients underwent lobectomy, isthmectomy, or the combination of lobectomy and isthmectomy. Most histological diagnoses were benign (85.42%). The submental endoscopic thyroidectomy group showed better scores in the energy/fatigue, emotional wellbeing, and general health domains (*p* = 0.006, 0.041, and 0.004, respectively). There were no statistically significant differences in surgical outcomes between the submental endoscopic thyroidectomy and conventional thyroidectomy groups (*p* > 0.05). Submental endoscopic thyroidectomy is feasible, and permits a better quality of life in terms of the energy/fatigue, emotional wellbeing, and general health domains.

## 1. Introduction

Submental endoscopic thyroidectomy is one of the alternative approaches in some situations in which transoral endoscopic thyroidectomy is not feasible, such as when the tumor is larger than 5 cm, or the patient cannot accept the risk of wound infection or submental nerve injury.

The three-port submental endoscopic approach and its variations have been gradually gaining acceptance since we introduced these approaches in 2016 [1,2,3]. The advantages of this method include the following: (1) no tumor size limit; (2) no paresthesia around the nipples, chest wall, or chin; (3) the ability of the midline endoscopic view to provide a better orientation from which surgical landmarks are more visible; (4) the ability to perform bilateral thyroid and parathyroid dissection; and (5) scars being hidden when the neck is in a natural position [4,5].

To date, the quality of life among patients undergoing submental endoscopic thyroidectomy has not yet been investigated. Thus, the objective of this study was to compare the surgical outcomes and quality of life between patients undergoing submental endoscopic thyroidectomy and conventional thyroidectomy.

## 2. Materials and Methods

### 2.1. Study Design and Setting

This prospective cohort study was conducted from January 2017 to December 2020 at the Department of Otorhinolaryngology, Faculty of Medicine, Khon Kaen University, Thailand.

### 2.2. Patient Enrollment

The patients were allowed to choose whether they would undergo traditional transcervical thyroidectomy or endoscopic thyroidectomy. In our institute, we offered the submental, transoral, and transaxillary approaches to endoscopic thyroidectomy.

After a discussion of the risks and benefits of each technique, the patients who chose the traditional and submental endoscopic thyroidectomy approaches were asked to participate in this study.

### 2.3. Operative Method Selection

After recruitment, the patients were given the information on the study and asked for informed consent. Participation in this study was voluntary, and the participants were free to withdraw at any time. All patients enrolled in the study signed an informed consent form to participate.

The patients were allowed to spend time to discuss with their family whether they would undergo endoscopic submental thyroidectomy or conventional thyroidectomy.

The selection of the operative method was left to the patient. The physicians respected the patients’ autonomy and decision of the type of surgery.

### 2.4. Surgical Techniques

**Figure 1:**
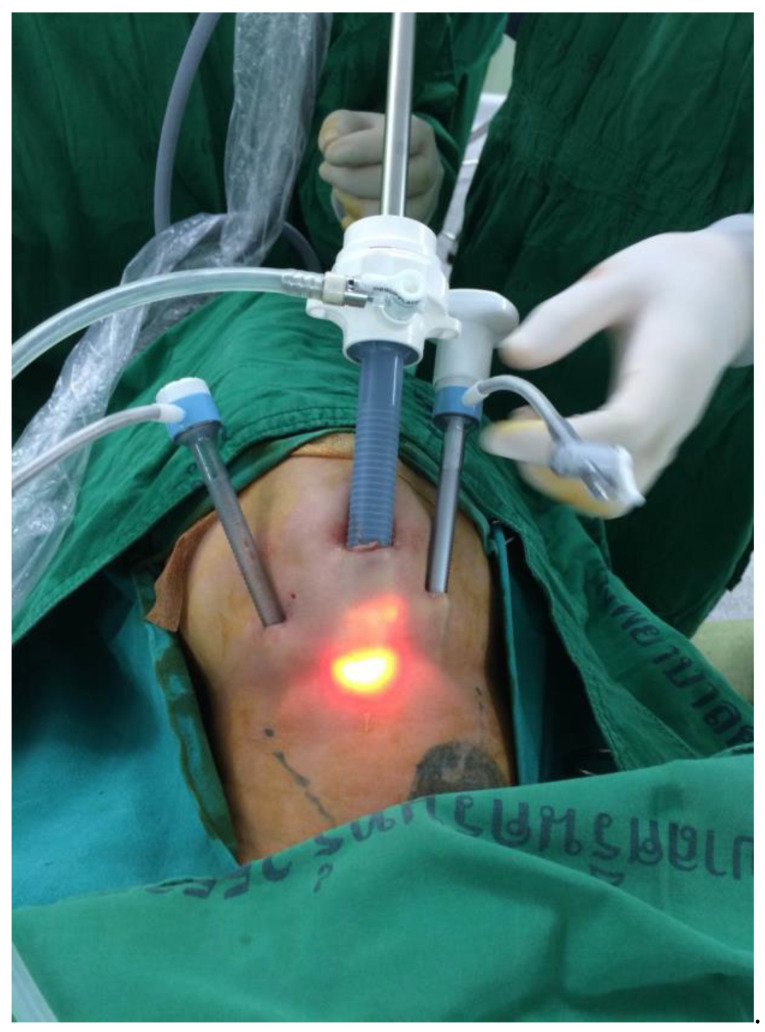
Submental endoscopic thyroidectomy.

For submental endoscopic thyroidectomy, the details of the surgical technique are described in our previous studies. The main difference from transoral thyroidectomy is the placement of the ports. The first midline mini-incision for a 10 mm port is made in the submental area between the chin and the hyoid bone. Bilateral submental incisions are then made for the 5 mm short trocars. At least two fingerbreadths below the mandible are recommended to avoid injury to the submental nerve [1,2,3] (Figure 1).

The surgical technique for conventional thyroidectomy is detailed elsewhere [6].

### 2.5. Quality-of-Life Assessment

The quality of life was assessed using the SF-36 questionnaire and our specific thyroid questionnaire at 2, 6, 12, and 24 weeks postoperatively.

The SF-36 questionnaire is a multipurpose short-form health survey with only 36 questions. The questionnaire evaluates 8 domains, including physical functioning (10 items), bodily pain (2 items), role limitations due to physical health problems (4 items), role limitations due to personal or emotional problems (4 items), emotional wellbeing (5 items), social functioning (2 items), energy/fatigue (4 items), and general health perceptions (5 items). The scores for each domain range from 0 to 100, with a higher score indicating a more favorable health state [7,8].

### 2.6. Surgical Outcomes Questionnaire

The specific questionnaire included 10 items. Overall satisfaction and visual analog scale (VAS) pain score at the operation site were graded from 0 (no pain) to 10 (worst pain ever) [9]. The tingling sensation and paresthesia, neck movement, shoulder movement, voice, swallowing, and cosmetic satisfaction were evaluated according to the subjective assessment of patients, on a scale from 0 (worse, always experience this problem) to 10 (excellent, no problem) [10].

### 2.7. Data Collection

The data were collected from outpatient and inpatient records, along with our predefined case record form. The clinical diagnosis, operative technique, pathological results, surgical outcomes, quality of life, and complications were recorded.

### 2.8. Statistical Analysis

Statistical analysis was conducted using SPSS (IBM, Armonk, NY, USA). Continuous variables were analyzed using the unpaired *t*-test or the Mann–Whitney U test for continuous variables and Pearson’s χ^2^ test or Fisher’s exact test for categorical variables, as appropriate, to analyze the statistically significant differences in the potential risk factors between the two groups. For repeated measurements of outcomes, the repeated-measures ANOVA was used. For all tests, *p* < 0.05 was considered to be statistically significant.

### 2.9. Ethics

Approval was sought from the Khon Kaen University Ethics Committee for Human Research before initiating the study (HE591517). All procedures performed in studies involving human participants were in accordance with the ethical standards of the institutional and/or national research committee, as well as with the 1964 Declaration of Helsinki and its later amendments or comparable ethical standards.

## 3. Results

Forty-eight patients were included in the study. Nine patients were male, and thirty-nine patients were female. Their ages ranged from 20 to 60 years. The mean duration of symptoms was 2 years. The mean body mass index (BMI) for both groups was within normal weight status. Some patients (12.50%) had underlying diseases; however, there were no statistically significant differences between groups in this regard (Table 1).

All patients underwent lobectomy, isthmectomy, or the combination of lobectomy and isthmectomy. Most histological diagnoses were benign (85.42%). There were no significant differences in nodule size, operative time, or complications between groups (Table 2).

The pain levels were mild (0–3 points) in both groups, and the pain scores decreased over time. There were no significant differences in pain scores between the groups (*p* = 0.146). Problems such as tingling/paresthesia, vocal changes, impaired swallowing, neck movement impairment, and shoulder movement impairment were mild (8–10 points), and there were no significant differences between the groups (*p* > 0.05). The levels of cosmetic satisfaction were moderate (4–7 points) in both groups. There were no significant differences in cosmetic satisfaction scores between the groups (*p* = 0.220) (Table 3).

The SF-36 scores were increased from baseline in all eight domains for both groups. The submental endoscopic thyroidectomy group showed better scores in the energy/fatigue, emotional wellbeing, and general health domains (*p* = 0.006, 0.041, and 0.004, respectively) (Table 4).

## 4. Discussion

The endoscopic thyroidectomy was developed with the main objective of improving cosmetic results. Scarless techniques such as transoral approaches are the most popular techniques for adolescents and young adults in our institute. However, these techniques are limited by factors such as tumor size and risk of submental nerve injury. Since 2016, we have introduced submental endoscopic thyroidectomy as an optional approach to overcome these problems [3].

In a previous study, we found that cosmetic outcomes and overall satisfaction were significantly better in the transoral endoscopic thyroidectomy group than in the conventional surgery group at all follow-up times (*p* < 0.05). The quality of life was also better in terms of the physical activity, psychosocial impairment, and physical and emotional domains [10].

Although submental endoscopic thyroidectomy and its variations have been adopted in many institutes [11,12], to the best of our knowledge, this is the first prospective cohort study to compare surgical outcomes and quality of life between submental endoscopic thyroidectomy and conventional thyroidectomy.

### 4.1. Operating Times

The new surgical technique takes time to learn. In our previous studies, the mean operating time required for both the transoral and submental approaches ranged from 120 to 180 min. However, in this study, the mean operating time was reduced to around 110 min, and there was no statistically significant difference from conventional thyroidectomy (*p* > 0.05).

This result may imply that when the surgeon reaches the plateau of the learning curve, the operating time for endoscopic approaches may be comparable to that of conventional approaches.

### 4.2. Cosmetic Satisfaction

Submental endoscopic thyroidectomy leaves small scars, and is therefore less cosmetically preferable when compared with the scarless transoral approach. However, in the event that the transoral approach is not feasible—such as with large tumors—small scars from alternative approaches are unavoidable.

In this study, we compared the cosmetic satisfaction between the submental approach and conventional thyroidectomy. The cosmetic satisfaction for the submental endoscopic thyroidectomy was greater than that for the conventional thyroidectomy; however, the difference did not reach statistical significance.

This result suggests that the submental endoscopic thyroidectomy may not be the best choice for patients who are concerned about their appearance and scars.

### 4.3. Complications

The recurrent laryngeal nerve injury was the most common complication for all types of thyroidectomy. Although there were no major complications in this study, we would still like to emphasize that the meticulous dissection of the thyroid capsule is the technique used to prevent this injury in both the endoscopic and conventional approaches.

### 4.4. Quality of Life

The submental endoscopic thyroidectomy group showed better scores in the energy/fatigue, emotional wellbeing, and general health domains (3/8 domains). These results could confirm that the minimally invasive nature of endoscopic thyroidectomy leads to more rapid recovery and return to normal life.

There was a discordant drop in quality-of-life scores in the energy/fatigue and general health domains at 12 weeks after surgery. This may have influenced the final statistical results. In the emotional wellbeing domain, a clearer trend in favor of submental endoscopic thyroidectomy was observed.

One SF-36 domain had statistically significant differences at baseline, i.e., physical functioning; this may have influenced the statistical results.

The minimal clinically important differences in SF-36 scores have been studied in other conditions, such as orthopedic surgery [13] and rheumatology [14]. There has been no direct study of minimal clinically important differences in thyroid diseases. However, a general minimal clinically important difference score of 5–10 has been used in several thyroid carcinoma studies [15,16,17].

In our study, of the three domains that had statistically significant differences—energy/fatigue, emotional wellbeing, and general health—only two domains (emotional wellbeing and general health) had score differences greater than 5, indicating clinically important differences.

In our previous study comparing transoral endoscopic thyroidectomy and conventional thyroidectomy, we found that 5/8 domains of the SF-36 were better in the transoral endoscopic thyroidectomy group, including role limitations due to physical health, energy/fatigue, social functioning, emotional wellbeing, and role limitations due to emotional problems. However, in this study, we found that only 3/8 domains were statistically significant. The discrepancy between these two studies was mainly in the emotional domains; we might therefore infer that scarless techniques such as transoral endoscopic thyroidectomy lead to better emotional wellbeing.

### 4.5. Limitations

This study’s sample was relatively small. To obtain more concrete evidence, we suggest that a larger, multicenter study is warranted.

## 5. Conclusions

Submental endoscopic thyroidectomy is feasible, and permits a better quality of life in terms of the energy/fatigue, emotional wellbeing, and general health domains. The cosmetic satisfaction with submental endoscopic thyroidectomy was higher than that with conventional thyroidectomy; however, this difference did not reach statistical significance.

## Figures and Tables

**Table 1:** Demographic data.

	Submental Endoscopic Thyroidectomy (*n* = 24)	Conventional Thyroidectomy (*n* = 24)	*p*-Value
Sex (male: female)	5:19	4:20	0.731 ^a^
Age (years)	41.08 ± 11.22	43.06 ± 11.19	0.576 ^b^
Duration of symptoms (years)	2.67 ± 4.32	2.70 ± 2.62	0.978 ^b^
Underlying diseases			
- Thalassemia	2	0	0.489 ^a^
- Allergic rhinitis	1	0	0.999 ^a^
- Hypertension	1	2	0.617 ^a^

^a^ Fisher’s exact test; ^b^ independent *t*-test.

**Table 2:** Operative details.

	Submental Endoscopic Thyroidectomy (*n* = 24)	Conventional Thyroidectomy (*n* = 24)	*p*-Value
Procedure			
- Lobectomy	20	24	0.109 ^a^
- Isthmectomy	2	0	0.489 ^a^
- Lobectomy and Isthmectomy	2	0	0.489 ^a^
Histological diagnosis			
- Thyroiditis	2	1	0.999 ^a^
- Multinodular goiter	4	6	0.724 ^a^
- Graves’ disease	0	1	0.999 ^a^
- Thyroid cyst	2	0	0.489 ^a^
- Follicular adenoma	13	11	0.773 ^a^
- Hurthle cell adenoma	0	1	0.999 ^a^
- Follicular carcinoma	1	0	0.999 ^a^
- Papillary carcinoma	2	3	0.999 ^a^
- Hurthle cell carcinoma	0	1	0.999 ^a^
Maximum nodule size (cm.)	2.87 ± 0.46	3.29 ± 1.01	0.077 ^b^
Operative time (minutes)	109.91 ± 53.65	113.79 ± 32.31	0.809 ^b^
Complications			
- Vocal cord paresis/paralysis	0	0	0.999 ^a^
- Surgical site infection	0	0	0.999 ^a^

^a^ Fisher’s exact test; ^b^ independent *t*-test.

**Table 3:** Results of the surgical outcomes questionnaire.

Scale (0–10)	Submental Endoscopic Thyroidectomy (*n* = 24)	Conventional Thyroidectomy (*n* = 24)	Mean Difference (95% CI)	*p*-Value ^a^
Pain	0 = No pain, 10 = Worst pain			
- 2 weeks	1.05 ± 2.38	1.31 ± 1.96	−0.26 (−1.75 to 1.22)	0.146
- 6 weeks	0.56 ± 1.96	0.22 ± 0.67	0.34 (−1.72 to 1.18)	
- 12 weeks	0.27 ± 0.65	0.50 ± 0.71	−0.23 (−1.33 to 0.88)	
- 24 weeks	0.14 ± 0.38	0.50 ± 0.71	−0.36 (−1.12 to 0.48)	
Tingling/paresthesia	0 = Always experience this problem, 10 = No problem			
- 2 weeks	9.29 ± 1.27	9.31 ± 1.45	−0.02 (−0.94 to 0.88)	0.485
- 6 weeks	9.31 ± 1.01	9.67 ± 0.71	−0.36 (−1.15 to 0.44)	
- 12 weeks	9.64 ± 0.67	10.00 ± 0.00	−0.36 (−1.45 to 0.72)	
- 24 weeks	9.57 ± 0.79	10.00 ± 0.00	−0.43 (−1.81 to 0.95)	
Vocal changes	0 = Always experience this problem, 10 = No problem			
- 2 weeks	8.90 ± 2.47	9.50 ± 1.10	−0.60 (−1.94 to 0.75)	0.419
- 6 weeks	8.31 ± 2.70	9.78 ± 0.67	−1.47 (−3.38 to 0.45)	
- 12 weeks	8.73 ± 2.19	10.00 ± 0.00	−1.27 (−4.81 to 2.27)	
- 24 weeks	9.14 ± 2.27	10.00 ± 0.00	−0.86 (−4.84 to 3.12)	
Impaired swallowing	0 = Always experience this problem, 10 = No problem			
- 2 weeks	8.76 ± 1.97	9.25 ± 1.48	−0.49 (−1.69 to 0.71)	0.221
- 6 weeks	9.25 ± 1.24	9.67 ± 1.00	−0.42 (−1.42 to 0.58)	
- 12 weeks	9.55 ± 1.04	10.00 ± 0.00	−0.45 (−2.13 to 1.22)	
- 24 weeks	9.29 ± 1.25	10.00 ± 0.00	−0.71 (−2.92 to 1.49)	
Neck movement impairment	0 = Always experience this problem, 10 = No problem			
- 2 weeks	8.57 ± 1.83	9.50 ± 0.89	−0.93 (−1.94 to 0.08)	0.257
- 6 weeks	9.38 ± 0.96	9.56 ± 1.01	−0.18 (−1.02 to 0.66)	
- 12 weeks	9.82 ± 0.41	10.00 ± 0.00	−0.18 (−0.83 to 0.47)	
- 24 weeks	9.86 ± 0.38	10.00 ± 0.00	−0.14 (−0.81 to 0.52)	
Shoulder movement impairment	0 = Always experience this problem, 10 = No problem			
- 2 weeks	6.71 ± 0.64	6.75 ± 0.45	−0.04 (−0.42 to 0.35)	0.267
- 6 weeks	6.88 ± 0.33	7.00 ± 0.00	−0.12 (−0.35 to 0.11)	
- 12 weeks	6.82 ± 0.41	7.00 ± 0.00	−0.18 (−0.83 to 0.47)	
- 24 weeks	6.88 ± 0.35	7.00 ± 0.00	−0.12 (−0.73 to 0.48)	
Cosmetic satisfaction	0 = Worst, 10 = Excellent			
- 2 weeks	6.52 ± 0.75	6.44 ± 0.89	0.08 (−0.46 to 0.64)	0.220
- 6 weeks	6.59 ± 0.71	6.44 ± 0.73	0.15 (−0.47 to 0.75)	
- 12 weeks	6.64 ± 0.51	6.00 ± 1.41	0.64 (−0.45 to 1.72)	
- 24 weeks	6.88 ± 0.35	6.00 ± 1.41	0.88 (−0.22 to 1.97)	

^a^ Repeated-measures ANOVA.

**Table 4:** General quality-of-life parameters.

Scale (0–100)	Submental Endoscopic Thyroidectomy (*n* = 24)	Conventional Thyroidectomy (*n* = 24)	Mean Difference (95% CI)	*p*-Value ^a^
Physical functioning				
- Baseline	90.91 ± 12.50	81.47 ± 15.29	9.44 (0.43 to 18.45)	0.798
- 2 weeks	91.19 ± 9.99	79.69 ± 17.08	11.50 (2.42 to 20.59)	
- 6 weeks	82.35 ± 26.05	92.22 ± 10.64	−9.87 (−28.70 to 8.97)	
- 12 weeks	93.18 ± 11.89	82.50 ± 24.75	10.68 (−12.28 to 33.64)	
- 24 weeks	93.13 ± 8.84	100.00 ± 00.00	−6.88 (−21.95 to 8.20)	
Role limitations due to physical health				
- Baseline	79.55 ± 27.43	63.24 ± 36.57	16.31 (−4.43 to 37.05)	0.533
- 2 weeks	76.19 ± 39.11	62.50 ± 42.82	13.69 (−13.76 to 41.14)	
- 6 weeks	76.47 ± 40.96	72.22 ± 44.10	4.25 (−31.51 to 40.01)	
- 12 weeks	86.36 ± 25.89	50.00 ± 70.71	36.36 (−18.83 to 91.55)	
- 24 weeks	81.25 ± 25.88	50.00 ± 70.71	31.25 (−32.19 to 94.69)	
Role limitations due to emotional problems				
- Baseline	75.76 ± 35.90	78.43 ± 33.22	−2.67 (−25.41 to 20.08)	0.566
- 2 weeks	79.37 ± 37.23	74.99 ± 31.05	4.38 (−19.01 to 27.76)	
- 6 weeks	72.55 ± 39.50	66.67 ± 44.10	5.88 (−29.08 to 40.85)	
- 12 weeks	90.91 ± 21.56	83.35 ± 23.55	7.56 (−29.24 to 44.36)	
- 24 weeks	87.50 ± 24.81	83.35 ± 23.55	4.15 (−40.79 to 49.09)	
Energy/fatigue				
- Baseline	75.23 ± 18.42	66.47 ± 16.65	8.76 (−2.81 to 20.32)	0.006 *
- 2 weeks	70.24 ± 23.90	72.50 ± 10.33	−2.26 (−15.26 to 10.73)	
- 6 weeks	73.82 ± 18.67	77.22 ± 17.52	−3.40 (−18.96 to 12.17)	
- 12 weeks	86.36 ± 8.69	57.50 ± 10.61	28.86 (13.84 to 43.88)	
- 24 weeks	81.88 ± 19.26	80.00 ± 14.14	1.88 (−32.21 to 35.96)	
Emotional wellbeing				
- Baseline	77.64 ± 14.43	71.29 ± 11.77	6.35 (−2.39 to 15.07)	0.041 *
- 2 weeks	79.81 ± 14.94	84.75 ± 11.52	−4.94 (−14.09 to 4.21)	
- 6 weeks	77.65 ± 18.66	84.44 ± 9.48	−6.79 (−20.57 to 6.97)	
- 12 weeks	86.18 ± 11.64	82.00 ± 14.14	4.18 (−15.94 to 24.30)	
- 24 weeks	87.50 ± 10.13	82.00 ± 14.14	5.50 (−14.03 to 25.03)	
Social functioning				
- Baseline	84.66 ± 21.45	69.12 ± 26.92	15.54 (−0.14 to 31.23)	0.948
- 2 weeks	86.31 ± 20.88	82.81 ± 22.30	3.50 (−10.99 to 17.98)	
- 6 weeks	80.88 ± 26.19	83.33 ± 25.77	−2.45 (−24.61 to 19.71)	
- 12 weeks	89.77 ± 13.48	100.00 ± 00.00	−10.22 (−31.98 to 11.52)	
- 24 weeks	78.13 ± 33.91	93.75 ± 8.84	−15.62 (−73.72 to 42.47)	
Pain				
- Baseline	82.73 ± 17.86	71.91 ± 26.47	10.82 (−3.58 to 25.21)	0.182
- 2 weeks	84.52 ± 18.02	79.22 ± 21.11	5.30 (−7.77 to 18.38)	
- 6 weeks	85.88 ± 15.69	91.67 ± 10.53	−5.79 (−17.85 to 6.28)	
- 12 weeks	97.05 ± 9.80	50.00 ± 38.89	47.05 (21.68 to 72.41)	
- 24 weeks	85.31 ± 18.10	95.00 ± 7.07	−9.69 (−40.89 to 21.51)	
General health				
- Baseline	67.95 ± 19.74	61.18 ± 18.67	6.77 (−5.84 to 19.40)	0.004 *
- 2 weeks	74.29 ± 17.84	70.94 ± 19.08	3.35 (−9.03 to 15.73)	
- 6 weeks	71.47 ± 22.96	70.56 ± 23.11	0.91 (−18.66 to 20.49)	
- 12 weeks	81.36 ± 12.27	45.00 ± 28.28	36.36 (11.87 to 60.85)	
- 24 weeks	81.25 ± 14.08	67.50 ± 3.54	13.75 (−10.37 to 37.87)	

^a^ Repeated-measures ANOVA, * statistically significant.

## Data Availability

Not applicable.
